# Author Index for Volume 99

**DOI:** 10.1038/sj.bjc.6604831

**Published:** 2008-12-09

Aapro, M 14

Abbate, R 225

Abbruzzese, JL 722

Adami, H-O 527, 1544

Adamietz, IA 1579

Adams, GP 1415

Adrian, TE 1064

Agnanti, N 2094

Agrawal, RK 1226

Ahern, TP 616

Ahmed, M 57

Ahn, MJ 167

Ahn, YC 167

Ait Ouakrim, D 1958

Ajani, JA 1195

Akagi, T 781

Akiba, S 408

Akil, N 404

Akleyev, A 1940

Al Moustafa, A-E 404

Al Murri, AM 1013

Albano, L 225

Albero, G 230

Alessandroni, P 716, 1402

Alexander, BH 545

Allen, CJ 2037

Alon, N 1129

Al-Saad, S 1476

Al-Shibli, K 1476

Alston, RD 830

Altés, A 1050

Altman, DG 883

Ambroisine, L 314

Ambros, PF 1027

Amin, T 72

Ammar, AA 327

Amonkar, MM 711, 1572

Anderson, M 100

Andersson, S 563

Ando, Y 655

André, F 68

André, T 862

Ang, JE 1365

Angelillo, IF 225

Anjomshoaa, A 966

Anson, K 1040

Anthony, L 722

Antonini, NF 275, 1316

Antoniou, AC 974

Aoki, D 1651

Apel, A 622

Arai, Y 1891

Aravantinos, G 1775

Argilés, JM 2029

Arisio, R 1357

Arkenau, H-T 1365

Armaroli, P 239

Armel, S 371

Armstrong, GR 459

Arnold, N 974

Arribas, J 1607

Arrieta, O 160

Arun, B 68

Asamura, H 852

Asanuma, T 1442

Aschroft, L 442

Ashley, DM 294

Ashraf, SQ 789, 839

Ashton, SE 1256

Astolfi, A 1729

Atanackovic, D 930

Atkinson, MJ 1089

Attard, G 314

Attard, NR 1276

Aubele, M 1089

Auer, G 513, 1089, 1121

Auperin, A 357

Austoker, J 1007

Autier, P 1185, 1958

Auvinen, A 182

Åvall-Lundqvist, E 1121

Avril, M-F 364

Awidi, AS 488

Ayers, M 68

Aytes, A 1718

Ayyaz, S 1176

Baasch, C 1269

Baas-Thijssen, MCM 875

Backen, A 841

Bäcklund, LM 1144

Baek, J-H 167

Baek, JH 584

Baer, HJ 1916

Bagnardi, V 1564

Baguley, BC 1678

Baiget, M 1050

Baixeras, N 1718

Baka, S 442

Baker, K 1712

Baker, MJ 1859

Baldelli, AM 1402

Baldi, G 716

Ballard-Barbash, R 1161

Balmaña, J 1607

Balzarini, J 481

Bandiera, E 768

Bang, Y-J 1593

Bankhead, C 1007

Banno, K 1651

Barathova, M 1348

Barbachano, Y 57

Bar-Eli, M 734

Barkauskas, DS 911

Barnadas, A 1050

Barrois, M 364

Bartels, K 930

Bartels, U 1129

Bartlett, JMS 341, 1089

Baselga, J 868, 1607

Bathers, S 1226

Batra, SK 520, 949

Bawohl, M 836

Baxter, D 1908

Baylin, SB 384

Bearz, A 51

Beckermann, BM 622

Beck-Popovic, M 1027

Bedikian, AY 734

Beer, H 1929

Begent, R 321

Begent, RHJ 632

Beglinger, C 1712

Bell, J 1013

Bellec, S 1191

Bellomi, A 239

Belshaw, NJ 136

Benetou, V 191

Bengochea, A 143

Benhadji, KA 1808

Benitez, J 974

Benner, A 1867

Ben-Shlomo, Y 1040

Benson, M 868

Benson, VS 185

Beral, V 185

Berbera, N 1586

Berger, W 151

Berglund, G 1975

Berkhof, J 259

Bernards, R 398

Berndsen, SC 481

Berney, D 314

Bertolini, F 1564

Bertrand, R 1613

Besarani, D 1383

Betton, B 1613

Bex, M 448

Bhattacharya, S 253

Bhide, SA 57

Bhogal, M 72

Bhogal, P 72

Bianco, R 473

Biasco, G 1729

Bichler, C 151

Bièche, I 2100

Bignotti, E 768

Billemont, B 1380

Billingham, LJ 883

Billings, LA 734

Binazzi, A 173

Birch, JM 830

Bird, SM 805

Birrer, MJ 2013

Bisonni, R 716

Bitzer, J 428

Bjørge, T 1165

Black, MA 966

Blackhall, F 442

Blair, A 1934

Blair, E 179

Blakey, DC 1256

Bleijenberg, G 1408

Blumenthal, RD 837

Boddy, AV 894

Bodmer, WF 789, 839

Boffetta, P 191, 207, 1912

Boige, V 1395

Boisdron-Celle, M 1239

Boitier, F 364

Bokemeyer, C 930

Bombled, J 364

Bonardi, R 539

Bonetti, F 675

Book, K 37

Bordoni, A 800

Borlak, J 1635

Bosch, A 455

Bosch, FX 230

Bouffet, E 1129

Boven, E 259

Bowen, RL 988

Bower, C 179

Bowman, A 597

Boyd, NF 1369

Boyle, PJ 1192

Bozec, A 93, 1556

Brabin, L 1908

Bradburn, DM 136

Brain, K 1007

Bramhall, SR 883

Brandberg, Y 1975

Brandi, G 1729

Brandl, T 37

Brandt, BH 774

Brard, L 1823

Braselmann, H 1089

Bratslavsky, G 1748

Braunstein, GD 781

Bremnes, RM 1476

Brennan, P 207, 1912

Brenner, H 133, 532, 1367

Bressac-de Paillerets, B 364

Brewer, D 1849

Briasoulis, E 1775

Bright, JJ 2044

Broderick, P 2088

Broecker, V 2065

Brown, MD 1859

Brown, NJ 1961

Brücher, B 1020

Brundler, M-A 1136

Brunstein, M-C 93, 1556

Brunt, AM 1226

Brunton, V 1769

Bruzzi, P 1027

Bryant, PE 670

Bucana, C 1426

Buchholz, M 1900

Buchholz, S 1246

Büchler, MW 622, 1064

Büchler, P 622

Buck, M 491

Budiningsih, S 214

Buechert, M 1579

Buerger, H 774

Buffa, FM 1884

Buffart, TE 1802

Buffler, P 1668

Bugat, R 1959

Bunce, CM 781

Burger, HU 14

Burger, M 78

Burger, MJ 491

Burgess, CC 1221

Burniat, A 1874

Busund, L-T 1476

Butler, LM 1511

Buzdar, AU 68

Byrne, GJ 1000

Cafferty, FH 539

Cai, L 2070

Cai, Z 1656

Calderone, TL 1265

Califano, R 442

Calleri, A 1564

Calvo, F 1808

Calza, S 768

Camacho, LH 734

Cameron, D 711

Cameron, DA 1984

Camidge, DR 1984

Campbell, EJ 1769

Canestrari, E 716

Cantwell, MM 434, 796, 1170

Cao, Y 930

Capella, G 1718

Caplin, ME 72

Cappuzzo, F 51, 83

Caputo, R 473

Cardillo, A 1564

Cardwell, CR 434, 1170, 1529

Carlsen, SM 201

Carmeliet, P 448

Carnaghi, C 83

Carraway, H 384

Carsin, A-E 266

Carter, BA 639

Carter, PJ 100

Carvalho, B 1802

Casabonne, D 185

Casiraghi, O 357

Castagneto, B 51

Castel, V 1027

Castillo, A 408

Castro, F 741

Catalano, G 1402

Catalano, V 716, 1402

Cavassini, M 800

Cawthorne, C 459

Céilleachair, AÓ 266

Ceresoli, GL 51

Ceribelli, A 51

Cerón-Lizarraga, TL 160

Cervantes, A 455

Chae, SW 1704

Chakraborty, S 520

Chambers, G 1908

Chambers, P 1276

Chan, C 375

Chan, FKL 2083

Chan, JK 1210

Chan, JY 2070

Chan, SY 774

Chander, SK 1433

Chandler, I 2088

Chang, HM 584

Chang, PN 1832

Chang, Q 1074

Chapman, R 1934

Charasson, V 1239

Charbonnier, F 551

Charles, AK 179

Charnsangavej, C 734

Chatelut, E 1239

Chaturvedi, P 949

Chearwae, W 2044

Chemin, I 143

Chen, C-H 1453

Chen, C-M 1453

Chen, E 1934

Chen, J 811

Chen, L-m 1210

Chen, P-P 1656

Chen, Z(G) 1684

Cheng, Y-M 1096

Cheng, YY 2083

Chiang, Y-C 23

Chie, L 1544

Chien, C-Y 1453

Chien, L-C 23

Chinegwundoh, F 1040

Chinnaswamy, G 894

Chiou, S-J 1453

Chirivella, I 455

Chiyoda, T 1651

Chompret, A 364

Chou, C-Y 1096

Chouaid, C 2006

Chow, W-H 1912

Christensen, O 1579

Christodoulou, C 1775

Chu, H 2001

Chuang, H-C 1453

Chuang, H-L 23

Chung, Y-H 1593

Chung, Y-J 1593

Ciardiello, F 473

Ciatto, S 239, 539

Clamp, AR 253

Clarhaut, J 1153

Clark, J 314

Clarke, NW 1859

Classen, J 1517

Clavel, J 1191

Clavien, P-A 836

Clemens, MR 862

Clements, A 1007

Cleven, AHG 727

Cliffe, AR 118

Clifford, GM 230, 800

Cold, S 604

Colditz, GA 995

Cole, M 894

Coleman, MP 219, 1923

Coleman, RE 597

Colleoni, M 1564

Collina, N 423, 1756

Collins, VP 1144

Colpaert, CG 1735

Comber, H 266

Conaghan, PJ 839

Conroy, T 1395

Consonni, D 239

Conte, M 1027

Cooke, TG 704, 1089, 1769

Cooper, CS 314, 1849

Cooper, N 1923

Cooper, WA 375

Corbishley, C 1040

Cormier, JN 734

Cornain, S 214

Cortés, A 1050

Cortinovis, D 51

Corvilain, B 1874

Couch, F 371

Coudert, B 1959

Coupe, A 136

Coyle, B 1136

Crabb, SJ 689

Craven, O 577

Crijns, APG 341

Crilly, M 1763

Cronin-Fenton, DP 266, 616

Crown, J 1644

Culine, S 1959

Cummings, J 841

Cummings, S 371

Cunningham, D 6, 868

Currey, N 375

Cuzick, J 314, 1572

Cvitkovic, E 1808

Cybulski, C 974

Dafni, U 1775

Dahse, R 90

Dainty, JR 136

Dal Maso, L 800

Daley, F 321

Dallimore, N 1929

Damiano, V 473

Daniele, G 473

Darnel, AD 404

Darnton, A 822, 1954

Datta, PK 957

Davi, D 239

Davies, MA 1265

Davies, S 1967

Davis, DW 734

Davis, FG 1940

Dawelbait, G 1484

De Backer, IC 30

De Bernardi, B 1027

de Bock, GH 341

de Boer, MA 214

de Bono, JS 314, 1365

de Bruïne, AP 727

de Gast, G 259

de Graeff, P 341

de Jong, S 341, 1600

de Klerk, N 179

De Kraker, J 1027

de Lacerda, AF 1027

de Lichy, M 364

De Lisi, V 423, 1756

de Rycke, Y 1027

de Sanjosé, S 230

de Souza, MM 143

De Souza, R 2037

de Vijver, MJ 1884

De Vincenzo, F 51

de Vries, EGE 341

Dean, E 841

Deane, NG 957

Deans, DAC 126

Dearling, JLJ 632

Debniak, T 974

Debus, J 900

Decallonne, B 448

Degen, L 1712

Del Conte, G 51

del Río, E 1050

Delord, J-P 1239

D’Emidio, S 1402

Deng, W 1265

Denton, J 383

Denz, A 1484

Desiderio, MA 1623

Destro, A 83

Detjen, K 110

Dewar, JA 1763

Di Fiore, F 551, 1586

Di Giuseppe, G 225

Di Tommaso, L 83

Diallo, J-S 1613

Diamandis, EP 1103, 1484

Diaz, M 230

Dibben, S 383

Dieckmann, K-P 1517

Diederichs, M 1517

Diehlmann, A 622

Dietel, M 939

Dieumegard, B 2100

Díez, O 974

Dillon, JF 805

Dimitriadi, M 1144

Dimopoulos, M-A 1775

Ding, X-Z 1064

Direcks, WGE 481

Dirix, LY 1735

Distler, M 1484

Dive, C 459, 841

Diwan, AH 734

Dobrovic, A 294

Dodson, AR 1849

Doh, ST 2070

Doki, Y 1307

Donnan, PT 1763

Donnem, T 1476

Doody, MM 545

Doran, P 1322

dos Santos Silva, I 1539

Dosaka-Akita, H 2013

Douchi, T 408

Doughty, JC 1013

Douillard, J-Y 1959

Dowlati, A 911

Downey, P 677

Drinkwater, KJ 695

Drummond, KJ 294

Du, R 911

Ducreux, M 1395

Dudley, AC 118

Duffy, MJ 1644

Duffy, SW 539, 557, 988, 1176

Dumez, H 448

Dumont, J-E 1874

Duniho, S 100

Dunn, JA 597, 1226

Durand, G 364

Durando, A 1357

Dzhelai, M 1251

Earl, HM 597, 1226

Economopoulos, T 1775

Eden, TOB 219, 830, 1967

Edwards, DR 126

Edwards, J 1296, 1769

Edwards, S 314

Eeles, R 1849

Ehret, C 415

Eisenbauer, M 151

Eizuru, Y 408

Ekbom, A 1544

EL-Khateeb, MS 488

Elliott, GO 136

Ellis, IO 327

Elmberger, G 1121

El-Rifai, W 949

Elsheikh, SE 327

Elzi, L 800

Engebjerg, MC 1522

Engel, C 974

Engel, J 1285

Engeland, A 1165

English, M 894

Epping, MT 398

Ercolani, G 1729

Erkan, M 760

Ernst, B 415

Eshtayeh, AA 488

Esposito, I 1064

Ess, S 800

Etienne-Grimaldi, M-C 93

Eton, O 734

Evans, S 1040

Eveno, C 1555

Evoy, D 1644

Ewertz, M 616

Faber, J 2029

Fahey, TP 1763

Fairley, L 1786

Faivre, S 1197, 1808

Faivre-Finn, C 442

Falchetti, M 768

Falcone, A 716

Falconer, A 1849

Fang, F-M 1453

Faried, A 1468

Farmer, D 1743

Farquharson, AL 591

Farshid, G 677

Fartoux, L 862

Favaretto, A 51

Fearon, KCH 126

Federico, M 423, 1756

Fee, BE 1136

Feldmann, G 1900

Feltbower, R 1967

Fendrich, V 1900

Feng, Z 1192

Fens, MHAM 1256

Fern, L 1967

Fernando, IN 597, 1226

Ferraldeschi, R 442

Ferrante, P 173

Ferretti, S 423, 1756

Fiander, AN 1929

Fidler, IJ 1426

Field, JK 557

Fielder, H 1929

Figueras, J 1718

Finarelli, AC 423, 1756

Findlay, M 1251

Finocchiaro, G 83

Fischel, J-L 93, 1556

Fisher, G 314

Fizazi, K 1959

Flask, C 911

Fletcher, A 314

Fletcher, JA 1600

Fleuren, GJ 214

Flohr, P 314

Foca, F 423, 1756

Fonck, M 1239

Fong, D 1290

Foot, A 1027

Foretova, L 1912

Forman, D 1786

Forman, MR 796

Fornaro, L 716

Foster, C 314

Foster, PA 1433, 1842

Foulkes, WD 371

Fountzilas, G 1775

Fouret, P 357

Foxall, RJ 136

Franc, B 1874

Franceschi, S 800

François, E 1395

Frank, H 44

Frazier, ML 734

Frebourg, T 551

Freemantle, N 883

Freemont, AJ 383

Freitag, L 2006

Frgala, T 1103

Friederichs, J 966

Friedman, HS 294

Friedrichs, K 930

Friend, PJ 1383

Friess, H 622, 760, 1064

Fritzsche, FR 939

Fröberg, M 563

Frommhold, D 622

Frost, A 1579

Frost, G 822, 1954

Fualal, J 63

Fujii, H 1462

Fujii, T 1651

Fujikane, T 384

Fujimori, H 655

Fujimoto, J 1557

Fujiwara, Y 1196, 1307

Fukai, Y 1468

Fukuchi, M 1468

Fukudo, S 176, 1502

Fukumoto, M 2020

Fukuyoshi, Y 655

Fukuzawa, R 966

Furneaux, CE 1678

Gabrielson, E 384

Gadzicki, D 974

Gajalakshmi, V 207

Gajapathy, S 83

Gakwaya, A 63

Gallo, F 239

Galukande, M 63

Galy, O 143

Gamelin, E 1239

Gan, HK 294

Ganesan, A 689

Gao, YT 811

Gardiner, RA 491

Gardner, P 1859

Garg, D 136

Garne, JP 616

Garrett, MD 1849

Garssen, J 2029

Gastl, G 1290

Gaujoux, S 1555

Gaya, A 321

Gazi, E 1859

Gaziano, JM 1743

Geinitz, H 1020

Gelardi, T 473

Gelderblom, H 275, 1316

Georgoulias, V 923

Geraci, M 830

Gerber, H-P 100

Gerner, C 151

Gershenwald, JE 734, 1265

Gerson, S 911

Geurts-Moespot, A 1644

Geyer, C 711

Ghabreau, L 404

Ghadirian, P 371

Ghaneh, P 6

Ghoul, A 1808

Gibbs, D 2006

Gielissen, MFM 1408

Giese, N 622, 1064

Giese, NA 760

Giese, T 760

Gill, PG 677

Gillatt, D 1040

Gillespie, LL 639

Ginsburg, OM 1369

Giordani, P 716, 1402

Giorgi-Rossi, P 239

Giovannetti, E 750

Giovannini, M 1395

Giustini, L 716

Glas, AM 398

Glimelius, B 1975

Goedendorp, MM 1408

Gogas, H 1775

Goldberg, DJ 805

Goldenberg, DM 837

Goldhirsch, A 1564

Goldie, SJ 230

Gonçalves, V 1794

Gonzalez, D 741

González-De la Rosa, CH 160

Gostner, JM 1290

Gout, PW 464

Grabe, N 1867

Grabsch, HI 1802

Gradin, K 1348

Graff, JR 750

Grant, J 2037

Grant, R 1967

Grasl-Kraupp, B 151

Gravekamp, C 741

Graziano, F 716, 1402

Grebenchtchikov, N 1644

Green, AJ 632

Green, AR 327

Green, J 185

Greene, MH 1748

Greither, T 1083

Greystoke, A 841

Grieve, RJ 1226

Grimaldi, MC 1556

Grimsley, S 1296

Grochola, LF 1083

Grosche, B 1946

Grossi, F 51

Groth, A 622

Grundy, RG 1136

Grützmann, R 1484

Guchelaar, H-J 275, 1316

Guevara-Salazar, P 160

Gui, D 781

Guilhot, J 1153

Guinebretière, JM 2100

Güth, U 428

Gutzmer, R 2065

Guzman, M 1607

Haanen, JBAG 259

Haapasalo, H 182

Haber, DA 1302

Habib, FK 126

Hainaut, P 143

Halder, SK 957

Hall, P 1544

Halmos, B 911

Hamann, U 974

Hamidou, H 1586

Hamilton-Dutoit, S 616

Hammel, P 1808

Hammerschmied, CG 78

Han, HY 1832

Han, J 167

Han, TQ 811

Hankinson, SE 1916

Hansen, HJ 837

Hara, A 647

Harding, A-H 822, 1954

Hardy, R 1539

Harrington, KJ 57

Harris, AL 1884

Hart, AAM 398

Hart, IR 988

Hartmann, A 78

Hartmann, JT 1517

Haruta, M 1891

Harvey, P 1226

Harwood, CA 1276

Hassan, AB 883

Hasumi, K 1216

Hatakeyama, D 647

Hauff, P 110

Haug, U 133, 532, 1367

Hawkins, C 1129

Hawkins, CJ 294

Hawkins, M 1967

Hayes, DN 683

He, X 1934

Hedley, DW 1074

Hegewisch-Becker, S 930

Heikkinen, T 974

Heilmann, M 900

Hein, S 1269

Helén, P 182

Helgason, HH 259

Hellbom, M 1975

Helley, D 1380

Helms, MW 774

Helsby, NA 1251

Hemminki, K 536

Henderson, BJ 1007

Henne-Bruns, D 1083

Hennig, M 1484

Hennig, R 1064

Henningson, M 1534

Hennink, WE 392, 900

Henriquez, FL 1673

Heo, DS 1593

Hernández-Pedro, N 160

Herr, I 622

Herschbach, P 37

Heuch, I 1165

Hey, Y 383

Hibbitts, S 1929

Hida, K 118

Higashi, M 408

Hildebrandt, Y 930

Hill, A 868

Hill, KJ 1256

Hiller, L 597, 1226

Hilmy, M 1013

Hirasawa, A 1651

Hirata, K 384

Hirsch, M 1984

Hirschel, B 800

Hirsimäki, P 335

Hirst, C 1572

Hisashige, A 1232

Hitzenbichler, F 78

Hiyama, E 1891

Hjerpe, A 563

Ho, AD 622

Hochhauser, D 72

Hodge, KM 1415

Hoefler, P 1867

Hoff, PM 722

Hoffbeck, RW 545

Hoffmeister, M 532

Höfler, H 1020

Hofmann, J 1368

Hofmann, W 974

Hofsli, E 1330

Hofstädter, F 415

Holcatova, I 1912

Hole, DJ 1046

Hollema, H 341

Holmberg, L 611

Holmes, AJ 83

Holotnakova, T 1348

Holzgreve, W 428

Holzmann, B 966

Holzmann, K 151

Homma, S 1034

Honda, S 1891

Hoover, RN 1161

Horak, E 1415

Horgan, G 1322

Horie, H 1891

Horlings, H 1884

Horn, C 930

Horne, E 1426

Horst, D 1285

Hortobagyi, GN 68

Horvath, L 375

Hosgood, HD 1934

Hosone, M 350

Hou, JM 841

Houlston, RS 2088

Houssami, N 539

Houston, A 502

Howard, HC 597

Howard, M 1136

Hozawa, A 1502

Hsieh, C-C 1161, 1544

Hsing, AW 811

Hsu, K-F 1096

Huang, C-C 1453

Huang, DJ 428

Huang, H-Y 1453

Huang, Y-F 1096

Huber, PE 900

Hudson, DL 1849

Humar, B 966

Hundt, S 133, 1367

Hunt, J 1684

Hurst, NG 555, 556

Hutchinson, SJ 805

Iavicoli, S 173

Ibrahim, F 1040

Ibrahim, JG 2001

Ichimura, K 1144

Ichinose, Y 852

Iida, K 2020

Iida, S 350

Im, S-A 1593

Imai, K 384

Inanami, O 1442

Ingleby, JD 986

Inose, T 1468

Inoue, K 176

Iossa, A 239

Ishibashi, M 2020

Ismail, SI 488

Ismail, T 556

Isobe, Y 1816

Issa, J 1256

Iwanicki-Caron, I 1586

Iwase, H 655

Jäger, C 939

Jain, M 520, 816

Jakubowska, A 974

James, R 1923

Jameson, MB 2006

Jänicke, F 1269

Jänne, PA 83, 245

Janot, F 357

Janout, V 1912

Janssen, CS 1673

Jayson, G 1794

Jensen, AØ 1522

Jensen, BV 858

Jensen, HA 858

Jeong, H-S 167

Jernström, H 1534

Jesser, C 1743

Jeziorska, M 383

Jhalli, R 1433

Jhavar, S 1849

Jiang, S 911

Jin, H 2083

Jin, L 1874

Jochum, W 836

Johansson, B 563, 1975

Johansson, U 1534

Johns, TG 294

Johnson, BE 245

Johnson, IT 136

Johnson, KJ 545

Johnson, MM 734

Johnson, PJ 883

Johnson, PWM 689

Johnston, PG 2054

Jombwe, J 63

Jonas, M 100

Jones, A 695

Jones, D 1426

Jones, J 1929

Jones, JL 988

Jono, H 655

Jonsson, H 1176

Jorgensen, C 1074

Judson, I 1365

Juhasz-Boess, I 1246

Jun, HJ 167

Jundt, G 800

Jung, A 1285

Jung, K 553, 939

Kabat, GC 816

Kakizaki, M 176, 1179, 1502

Kalikaki, A 923

Kallifatidis G 622

Kalluri, R 1375

Kalofonos, HP 1775

Kalogeras, KT 1775

Kalthoff, H 1484

Kamimura, K 1462

Kaminsky, M-C 1959

Kandioler, D 151

Kaneko, Y 1891

Kang, HJ 584

Kang, M 1668

Kang, WK 584

Kang, Y-K 584

Kanimozhi, V 207

Kannan, S 1340

Kantoff, P 1743

Kanyike, D 63

Kapp, A 2065

Kapp, DS 1210

Karagas, MR 1522

Karayan-Tapon, L 1153

Karran, P 1276

Kashiwakura, I 1442

Kassab, A 404

Katagiri, A 2020

Kataoka, F 1651

Kato, A 305

Kato, H 852, 1468

Kato, K 647

Katsaros, D 1357

Kavuma, A 63

Kawaguchi, Y 1462

Kawano, Y 663

Kawar, NM 1210

Kaye, AH 294

Kaye, SB 1365

Kegler, D 2029

Keh, C 556

Keir, M 894

Keku, TO 2001

Keller, M 37

Kelly, J 502

Kelly, JD 663

Kemming, D 774

Kemp, EG 1673

Kennedy, C 375

Kenward, MG 219

Kern, JA 911

Kester, AD 30

Khan, NA 408

Kiessling, F 900

Kigula-Mugambe, JB 63

Kijima, Y 408

Kikuchi, J 2013

Kilpatrick, MW 789

Kim, D-W 1593

Kim, HS 167

Kim, J 245

Kim, JG 584

Kim, JJ 230

Kim, JW 1593

Kim, KB 734

Kim, KK 1823

Kim, M 143

Kim, MA 1704

Kim, S 1302

Kim, S-J 1593

Kim, SH 741

Kim, T-W 584

Kim, T-Y 1593

Kim, WH 1704

Kim, Y 789

Kim, YJ 1593

Kimura, F 305

Kimura, H 1468

Kinoshita, I 2013

Kirby, R 1040

Kirchner, T 1285

Kirwan, CC 1000

Kitajewski, J 1204

Kitchener, H 1908

Klagsbrun, M 118

Kleeff, J 760

Klinkhammer-Schalke, M 415

Klip, HG 341

Kloor, M 1867

Kneitz, B 78

Knight, JF 1849

Knippschild, U 1083

Kobayashi, S 305

Kodaira, S 1232

Koeffler, HP 781

Koh, W-P 196, 1511

Kohonen-Corish, MRJ 375

Koike, T 852

Koivunen, JP 245

Kollarova, H 1912

Koller, M 415

Kong, A-N 2070

Kono, K 1462

Koopman, M 1316

Kopacek, J 1348

Kopetz, S 722

Korde, LA 1748

Koriyama, C 408

Korsching, E 774

Kosmehl, H 90

Kossenko, M 1940

Kotnis, A 1340

Koutras, AK 1775

Koutsopoulos, A 923

Kovacs, G 314, 1849

Kraetzschmar, J 1579

Krammel, C 1290

Krestinina, L 1940

Kreuzer, M 1946

Kriegl, L 1285

Krijgsman, O 398

Krishnamurthy, S 68

Krishnapuram, B 1884

Kristiansen, G 939

Kröger, N 930

Kroll, ME 1192

Kronqvist, P 335

Krupa, K 704

Kudo, M 655

Kuettler, U 2065

Kuh, D 1539

Kühnlein, R 900

Kumar, S 1000

Kunitoh, H 852

Kurek, R 868

Kuriyama, S 176, 1179, 1502

Kuwabara, M 1442

Kuwano, H 1468

Kweekel, DM 275, 1316

Kwon, J-H 1593

LaCroix, A 527

Lacroix, L 357

Ladenstein, R 1027

Lægreid, A 1330

Lafitte, J-J 2006

Lagergren, J 1506

Lagiou, P 191, 1544

Lahn, M 473

Lam, M 911

Lambert, LA 1114

Lambin, P 1884

Lammers, T 392, 900

Lammertsma, AA 481

Lan, L 513

Lan, Q 1934

Landers, KA 491

Langaas, M 1330

Lange, TS 1823

Langer, P 1900

Langleben, A 722

Lannigan, A 1013

Laplanche, A 1959

Larjavaara, S 182

Larsen, O 858

Lash, TL 616

Lassere, Y 722

Lattrich, C 1246

Laurand, A 1239

Laurent, M 551

Laureys, G 1027

Laurvick, CL 179

Laviano, A 2029

Lavin, MF 491

Lazar, AJF 1265

Le, PTK 1600

Le Page, C 1613

Le Pessot, F 551

Le Roux, E 143

Le Tourneau, C 1197, 1808

Lee, BL 1704

Lee, C-S 375

Lee, DTW 1832

Lee, HE 1704

Lee, H-J 1593

Lee, H-P 196

Lee, HS 1704

Lee, J 245

Lee, JE 734

Lee, J-L 584

Lee, K-H 584

Lee, K-M 1934

Lee, S-H 1593

Lee, SK 167

Lee, Y-C 1426

Lee, YJ 1704

Lee, Z 911

Leese, MP 1433, 1842

Lefebure, B 551

Lefrançois, L 143

Lein, M 939

Lenoir, GM 364

Lepage, C 1923

Lesimple, T 1959

Leslie, M 862

Lessard, L 1613

Lesueur, F 364

Leung, W-k 2083

Leuwer, R 930

Levillain, P 1153

Lewis, I 1967

Lewison, G 569

Leyland-Jones, B 1, 385

Li, H 1743

Li, L 513, 1103

Li, Q 1179

Lidereau, R 2100

Liedtke, C 774

Ligorio, C 83

Liguori, G 225

Lin, L 23

Lin, MC 283

Lin, S-H 1426

Lin, Y-H 966

Linardou, H 1775

Lindena, G 37

Linet, MS 545

Ling, MT 1832

Ling, V 464

Linsell, L 1221

Linton, KM 383

Lipton, L 1387

Lisnawati 214

Liu, L 911, 1144

Liu, Q 1695

Liu, X 2083

Llort, G 974

Lo, M 464

Lo, W-Y 1251

Löfdahl, HE 1506

Logothetis, CJ 1426

Loh, AXW 978

Lokiec, F 1808

Long, NK 647

Lordick, F 1020

Lorenz, W 415

Lorenzen, S 1020

Lorenzo Bermejo, L 536

Lorey, F 1668

Lorigan, P 442

Lortholary, A 1959

Loucaides, E 1967

Loud, JT 974

Loupakis, F 716

Louvet, C 862

Lovric, MM 294

Lowrie, AG 126

Lu, L 1357

Lu, Y 1506

Lubbe, S 2088

Luber, B 1089

Lubinski, J 974

Lüdde, R 1517

Ludwig, M 1579

Ludyga, N 1089

Luetkens, T 930

Lugli, A 1712

Luini, A 1564

Lunetta, KL 616

Luo, J 527, 1121

Luong, QT 781

Lüpfert, C 868

Luwaga, A 63

Lynch, HT 371

Lynge, E 1176

Ma, J 1129, 1743

Ma, PC 911

Ma, Q 1695

Maaser, K 1635

Macafee, DAL 1991

Maciag, PC 741

Mackey, JR 989

MacMathuna, P 1322

Maeda, S 350

Magdolen, V 1644

Magnani, M 716

Mahmoud, IS 488

Mahner, S 1269

Mai, PL 974

Mainwaring, PN 2006

Majdic, O 151

Makita, H 647

Makowski, L 683

Malkin, D 1129

Mallo, H 259

Maltese, P 716

Mancini, E 239

Mancuso, P 1564

Manda, S 1786

Manfrin, E 675

Mangone, L 423, 1756

Mansfield, PF 734

Marangolo, M 51

Marcuello, E 1050

Margolis, KL 527

Margreiter, R 1290

Marijnen, CAM 875

Marinaccio, A 173

Marks, JD 1415

Maroni, P 1623

Marquant, C 1153

Marshall, ES 1678

Marten-Mittag, B 37

Martens, JWM 1644

Martin, LJ 1369

Martinelli, F 539

Martinelli, G 1729

Martínez-Iniesta, M 1718

Martín-Martorell, P 455

Maruyama, T 1462

Marx, A 930

Mashima, T 283

Masi, G 716

Mason, P 1383

Massard, C 1959

Massie, CE 314

Mastrantonio, M 173

Mastuda-Ohmori, K 1179

Masuda, N 1468

Mates, D 1912

Mathers, JC 136

Mathew, A 207

Mathew, BS 207

Mathew, P 1426

Mathioudaki, K 2094

Matsubara, K 1307

Matsuda, A 1442

Matsuda-Ohmori, K 176

Matsumoto, K 1034

Matsutani, T 350

Matsuura, I 1816

Mattern, J 622

Matteucci, E 1623

Matveev, V 1912

Mavroudis, D 923

Maw, MK 1557

Maxwell, JA 294

Maxwell, P 2054

Mazouni, C 68

McAdam, K 1226

McArdle, CS 1046

McCall, JL 966

McCall, P 1296

McCann, R 1908

McCarthy, PL 639

McCaughan, B 375

McCollum, CN 1000

McConkey, DJ 734

McCormack, V 1539

McCowan, C 1763

McCoy, S 1387

McDermott, E 1644

McDonald, SA 805

McDowell, G 1000

McDuff, E 1769

McElvenny, D 822, 1954

McGregor, JM 1276

McGregor, SB 789

McGreivy, J 1387

McGuffog, L 974

McKay, IC 1673

McKeage, MJ 2006

McKeown, SR 2054

McKiernan, E 1644

McKinney, PA 1529

McMillan, DC 1013, 1046

McNicol, A-M 1013

McRea, J 1929

Medioni, J 1380

Meersma, GJ 1600

Mehmud, F 862

Meijer, C 1600

Meijer, GA 1802

Meijer, J 1493

Meindl, A 974

Menakuru, SR 1961

Mencoboni, M 51

Menzies, AR 1056

Mercer, FC 639

Meric, JB 1380

Merle, P 143

Merrouche, Y 1959

Mes-Masson, A-M 1613

Metcalfe, C 1040

Metges, JP 1395

Mews, PJ 1678

Meyer, T 72

Meyerson, M 245

Meza, JL 949

Miccinesi, G 423, 1756

Michalski, CW 760

Michel, P 551, 1586

Michel, S 1867

Michelakis, ED 989

Michon, J 1027

Micksche, M 151

Middleton, RJ 796

Mikeska, T 294

Mikulits, W 151

Milano, G 1, 93, 1556

Milde-Langosch, K 930, 1269

Miller, AB 816

Miller, CJ 383

Mills, GB 1265

Mills, IG 314, 663

Mills, PR 805

Milne, E 179

Min, YJ 584

Minaguchi, T 1034

Miot, F 1874

Mishra, G 1539

Mitsudomi, T 852

Mitterer, M 1290

Miyagi, Y 1651

Miyata, H 1307

Miyazaki, K 2020

Miyazaki, M 305

Miyazaki, T 1468

Mizukami, Y 1462

Moch, H 939

Moestikaningsih 214

Mohammad, HA 488

Mohapatra, G 1302

Mohr, T 151

Moldenhauer, G 622

Møller, H 611

Möller, P 1083

Molleví, DG 1718

Molls, M 1020

Molter, J 911

Molthoff, CFM 481

Monden, M 1307

Moniaux, N 520

Monteith, SJ 1678

Montgomery, DA 704

Moon, C 1743

Moore, LE 1912

Moore, PA 100

Morales-Espinosa, D 160

Moran, A 830

Morel, A 1239

Morgan, D 822, 1954

Mori, H 647

Mori, M 384

Moriyama, EH 2037

Morris, MJ 1426

Morrow, S 1726

Moser, P 1290

Moss, S 1991

Mosseri, V 1027

Mross, K 1579

Mu, K 513

Mu, L 1357

Mucci, L 1743

Mueller, C 1748

Mueller, N 800

Muir, G 1040

Mulherkar, R 1340

Mulholland, HG 434, 1170

Müller, S 1684

Munck-Wikland, E 1121

Murgo, AJ 734

Murphy, G 663

Murray, D 1322

Murray, LJ 434, 796, 1170, 1529

Murray, M 1251

Nagai, K 852

Nair, S 2070

Nakagawa, K 245, 852

Nakagawara, A 1891

Nakajima, M 1468

Nakaya, N 176, 1179, 1502

Nakayama, K 2020

Nakayama, N 2020

Naldoni, C 239, 423, 1756

Nam, R 371, 847

Nannini, M 1729

Naoki, K 245

Nargund, V 1040

Narod, SA 371, 847

Naska, A 191

Natsugoe, S 408

Neal, DE 663

Nekulova, M 1103

Neoptolemos, JP 6

Nesterova, A 100

Neuhausen, S 371

Neuwöhner, K 37

Nevalainen, O 335

Nevanlinna, H 974

Newbold, K 57

Newman, SP 1433, 1842

Newton, R 1383

Ng, CS 734

Ng, EKO 2083

Ng, W 978

Niederacher, D 974

Nielsen, KV 1296

Niendorf, A 930

Nijziel, MR 30

Nilsen, TIL 201

Nishikawa, N 384

Nishimura, M 2013

Nishimura, S 1651

Nishio, K 1757

Nojima, M 384

Nolan, L 689

Nomura, H 1651

Nomura, S 305

Noonan, A 72

Norman, AR 1849

Northover, JMA 1923

Nortier, JWR 1316

Ntouroupi, TG 789

Nur, U 1923

Nutting, CM 57

Nuyten, DSA 1884

Oates, J 868

O’Brien, K 1644

O’Callaghan, G 502

Odicino, FE 768

O’Donovan, P 1276

O’Dwyer, ST 459, 591

Ogbourne, SM 1808

Ogink, J 1493

Ogura, A 1442

Oh, D-Y 1593

Oh, WK 1426

Ohi, Y 408

Ohira, M 1891

Ohmori-Matsuda, K 1502

Ohmura, T 384

Ohradanova, A 1348

Ohtsuka, M 305

Oien, K 341

Oikonomopoulou, K 1103

Oizumi, S 2013

Okada, M 852

Okada, S 1034

Okamoto, I 245

Oki, A 1034

Olesen, AB 1522

Olmos, D 1365

Olopade, O 371

Olsen, AH 1176, 1549

Olsson, H 1534

Onuki, M 1034

Opdahl, S 201

O’Reilly, S 1226

Orfanos, P 191

Orlando, L 1564

O’Rourke, D 2054

Ortmann, O 1246

Osann, K 1210

Oshimi, K 1816

Oskouian, RJ 1678

Osman, T 1064

Osorio, A 974

Ostertag, H 2065

Ostroumova, E 1940

O’Sullivan, B 695

O’Sullivan, JM 2054

Ota, K 655

Ota, T 1216

Ott, K 1020

Ouakrim, DA 1185

Oudard, S 1380

Overmeer, RM 1802

Oyee, J 1984

Paci, E 423, 1756

Packham, G 689

Padhani, A 321

Padullés, L 1718

Paillot, B 1586

Pal, N 136

Palmer, K 577

Palshof, T 44

Pang, D 830

Panse, J 930

Pantaleo, MA 1729

Papadokostopoulou, A 2094

Papadopoulos, NE 734

Papakostas, P 1775

Paré, L 1050

Parent, N 1613

Park, JO 245

Park, K 167

Park, SR 584

Parkin, DM 1549

Parkkila, S 1348

Parmar, MKB 1923

Parry, A 894

Parsons, MFC 1433

Pastorek, J 1348

Pastorekova, S 1348

Patel, B 1040

Paterini, P 1729

Paterno, GD 639

Paterson, Y 741

Patterson, CC 1529

Pawson, T 1074

Peacock, J 2001

Péant, B 1613

Pearson, ADJ 1027

Pearson, DM 1144

Pecorelli, S 768

Pectasides, D 1775

Pedersen, L 616

Pedley, RB 321, 632

Peiffert, D 1395

Peissel, B 974

Penegar, S 2088

Pennington, CJ 126

Pepe, S 473

Pepper, SD 383

Pereira, SP 978

Perez, N 862

Perrett, CM 1276

Persad, R 1040

Persson, C 1975

Persson, M 1476

Peschel, C 1020

Peschke, P 900

Pession, A 1729

Pétain, A 1239

Peter, S 1712

Peterlongo, P 974

Peters, AAW 214

Peters, GJ 481, 750

Peters, MEJW 1408

Petersson, F 1121

Petersson, L-M 1975

Petraki, CD 1484

Petrella, M 423, 1756

Pettengell, R 253

Pfeiffer, P 858

Pfeiler, G 1246

Piazzi, G 1729

Pichlmeier, U 1517

Pieterse, AH 875

Pieterse, S 677

Pilarsky, C 939, 1484

Pineda-Olvera, B 160

Pinna, AD 1729

Piquette-Miller, M 2037

Pirie, K 185

Pirker, C 151

Pita, G 974

Pivot, X 1959

Pocard, M 1555

Poellinger, L 1348

Poirier, A-L 1239

Polanco-Echeverry, G 1726

Polesel, J 800

Poll, A 371

Polla, E 239

Pollán, M 974

Pombo-de-Oliveira, MS 1668

Pommerencke, T 1867

Ponnusamy, MP 520

Ponti, A 423, 1756

Poole, CJ 597, 1226

Popert, R 1040

Pospisil, H 774

Potenta, S 1375

Potiron, V 1153

Potischman, N 1161

Potter, BVL 1433, 1842

Poulogiannis, G 1144

Powe, D 327

Powell, CB 1210

Powell, N 1929

Pranesh, N 591

Prebble, E 1136

Preisler-Adams, S 974

Premkumar, A 1748

Preston, DL 1940

Preti, M 1357

Price, L 894

Price, TJ 1387

Prieto, VG 734, 1265

Priou, F 1959

Proby, CM 1276

Proost, N 481

Puliti, D 423, 1756

Punt, CJA 275, 1316

Purohit, A 1433, 1842

Pusztai, L 68, 385

Qu, P 2001

Quoix, E 2006

Qureshi, U 321

Rachakonda, G 957

Rachet, B 1923

Radford, JA 253, 383

Radice, P 974

Raguz, S 387

Rajagopalan, P 1579

Rajan, B 207

Rakha, EA 327

Ramaswamy, A 1900

Ramirez, AJ 1221

Ramos, E 1718

Rand, V 1136

Ranson, M 841

Rantanen, A 335

Rao, S 868

Rao, SM 1064

Rashid, A 811

Rasmussen, L 604

Ravaggi, A 768

Raymond, E 1197, 1808

Razis, E 1775

Rea, DW 1226

Reck, M 2006

Reed, MJ 1433, 1842

Reed, MWR 1961

Reeve, AE 966

Reid, A 314

Reis-Filho, JS 327

Rembao, D 160

Renehan, AG 459, 591

Rexin, A 110

Reyes-Vidal, JM 862

Richards, WG 245

Riches, AC 670

Richey, J 911

Rickenbach, M 800

Riddick, ACP 126

Ridley, L 1136

Ridolfi, E 1623

Ried, T 1121

Riener, M-O 836

Riera, J 1020

Riffkin, CD 294

Riggins, GJ 1136

Rikhof, B 1600

Riley, G 1251

Rimassa, L 83

Rinaldi, D 722

Ritchie, DM 597

Rixe, O 1380

Rizzo, S 1849

Robert, J 1239

Roberts, D 459

Roberts, F 1673

Roberts, ISD 1383

Roberts, SA 1908

Robertson, C 805

Robinson, D 611, 1754

Robinson, MK 1415

Roche, J 1153

Rodríguez-Braun, E 455

Roe, P 569

Roethling, N 1020

Rogers, AM 245

Rohan, TE 816

Romani, C 768

Romundstad, PR 201

Ron, E 1940

Roncalli, M 83

Ronco, G 239

Roos, E 1493

Roselló, S 455

Rosenberg, CL 616

Rosenberg, P 1748

Rosengren, RJ 1056

Rosenthal, MA 1387, 2006

Rosmorduc, O 862

Ross, JA 126, 545

Ross, MI 734

Ross, P 862

Rossi, D 1402

Rossi, E 83, 768

Rothman, N 1912

Rothmund, M 1900

Rouits, E 1239

Rouleau, E 2100

Rout, S 591

Rowan, S 830

Rowntree, C 1967

Roy, HK 949

Ruben, SM 100

Rubie, H 1027

Rückert, F 1484

Russeva, M 1415

Rustin, G 321

Rutka, J 1129

Ruzzo, A 716

Ryan, AJ 1256

Ryan, DA 988

Ryan, MC 100

Ryder, WDJ 253

Ryoo, B-Y 584

Ryott, M 1121

Ryu, M-H 584

Saad, F 1613

Saal, H 371

Saeb-Parsy, K 663

Saeger, H-D 1484

Saeger, W 1900

Saffrich, R 622

Sagara, Y 408

Sagmeister, S 151

Said, J 781

Saijo, N 852, 1757

Sakoda, LC 811

Sakurai, M 1034

Salamina, S 51

Salazar, J 1050

Salazar, R 1718

Salnikov, AV 622

Samaka, RM 327

Samaratunga, H 491

Sandler, RS 2001

Sankila, R 182

Sansur, CA 1678

Santin, AD 768

Santini, D 716, 1402

Santoro, A 51, 83

Sarin, R 1340

Sartori, C 760

Sasajima, K 350

Sasaki, F 1891

Sasako, M 1196

Sasieni, P 1549

Sato, Y 350

Satoh, T 1034

Satzger, I 2065

Sauliunaite, D 760

Saulnier, P 357

Saunders, E 383

Saunders, MP 459, 577, 591

Saura, C 1607

Savicky, MWJ 639

Scaltriti, M 1607

Scandlyn, MJ 1056

Scarano, E 1564

Scardino, P 314

Scarselli, A 173

Scells, B 491

Schaefer, T 2065

Scheithauer, W 862

Schep, G 30

Scherhag, A 14

Scheulen, ME 1579

Schiffelers, RM 1256

Schiffman, JD 1668

Schirner, M 110

Schlotter, CM 774

Schmutzler, RK 974

Schnelzer, M 1946

Schöffski, P 448

Scholefield, JH 1991

Scholz, A 110

Schønnemann, KR 858

Schötzau, A 428

Schröder, M 1484

Schubert, M 622

Schulte-Hermann, R 151

Schultheis, B 1579

Schulz, P 110

Schuster, T 1020

Schutte, B 727

Schwarz, J 1269

Scoazec, J-Y 143

Scorilas, N 2094

Scullin, P 2054

Seaton, A 2054

Sedano, L 1050

Seenath, MM 459

Segnan, N 239, 423, 1756

Segura-Pacheco, B 160

Seigneuric, R 1884

Seitz, JF 1395

Sekine, I 1757

Selektar, J 781

Senapati, S 949

Seng, TJ 375

Seppo, A 789

Serafini, M 239

Serke, M 2006

Serova, M 1197, 1808

Seth, A 847

Shago, M 1129

Shah, A 219

Shah, T 72

Shaller, CC 1415

Shanahan, F 502

Shanks, JH 1859

Sharma, P 949

Sharp, L 266

Sharples, KJ 1251, 1678

Shastri, YM 1366

Shchaveleva, I 1415

Shearer, J 1763

Shehata, M 151

Sheikh, HY 577

Shen, M 1934

Shen, MC 811

Shen, M-R 1096

Shen, S 1695

Shen, XJ 2001

Shepherd, CJ 1849

Sherrill, B 711, 1572

Shibata, T 647, 852

Shida, T 305

Shih, S-C 118

Shimizu, H 305

Shimizu, K 350

Shimizu, Y 2013

Shin, DB 584

Shin, DM 1684

Shin, HJC 1684

Shin, JY 1210

Shiniriki, S 655

Shinohara, M 655

Shinomura, Y 384

Shipley, J 1849

Sibbett, R 1673

Sieuwerts, AM 1644

Siewert, JR 1020

Sigmond, J 750

Sigona, A 423, 1756

Sigurdson, AJ 545

Silliman, RA 616

Silver, A 1726

Simickova, M 1103

Simmons, HH 1415

Simon, I 1103

Simon, SL 545

Simpson, A 1251

Sindrup, SH 604

Singh, AP 520, 1823

Singh, RK 1823

Siregar, B 214

Siu, JMT 2083

Sivathasan, N 72

Sjursen, A 577

Skipworth, RJE 126

Skokan, M 83

Slater, EP 1900

Sluss, P 1161

Smid, K 750

Smith, LM 100

Snijders, PJF 1802

Sogabe, Y 384

Sohda, M 1468

Soho, C 1748

Somers-Edgar, TJ 1056

Son, Y-I 167

Sone, T 176, 1179, 1502

Song, S 966

Sonoda, T 384

Sørensen, HT 616, 1522

Sørensen, JB 44

Sotelo, J 160

Souglakos, J 923

Spano, J-P 1

Spector, LG 545

Spells, K 1251

Spiertz, AJG 727

Spizzo, G 1290

Spooner, D 1226

Spreeuwenberg, MD 481

Spyratos, F 2100

Srirajaskanthan, R 72

Stahl, T 930

Stampfer, M 1743

Stanley, A 1226

Starling, N 6, 868

Starmans, MHW 1884

Stathopoulos, E 923

Staton, CA 1961

Steck, H 1884

Steenbergen, RDM 1802

Stefan, C 448

Stein, JM 1366

Stein, S 711, 1572

Steinbild, S 1579

Steinger, B 415

Stemke-Hale, K 1265

Stenning, S 1967

Stephan, C 939

Stephen, A 1539

Stewart, GD 126

Stiggelbout, AM 875

Stiller, CA 219, 1192

Stirling, JJ 321

Stocken, DD 556, 883

Stoehr, R 78

Stoppa-Lyonnet, D 371

Storm, G 392, 900, 1256

Stratford, IJ 459

Stratmann, R 760

Stretch, R 1908

Streubel, B 151

Strumberg, D 1579

Stuart, EC 1056

Su, C-Y 1453

Su, L 1684

Subr, V 900

Sudaka, A 93, 1556

Sugarbaker, DJ 245

Sughayer, MA 488

Sugimoto, K 1816

Sugiuchi, H 655

Sullivan, R 569, 2006

Sultana, A 6

Sun, P 371

Sun, S-Y 1684

Sun, Y-N 1387

Sun, Z-J 1656

Sundberg, ÅL 1415

Sundquist, J 536

Sundström, J 335

Sung, JJY 2083

Suraweera, N 1726

Susumu, N 1651

Sutherland, MK 100

Sutherland, RL 375

Suurmeijer, AJH 1600

Suzuki, A 1651

Suzuki, H 384

Suzuki, S 350

Swallow, DM 978

Sweep, FCGJ 1644

Swindell, R 383, 577, 591

Sydes, MR 1923

Szeszenia-Dabrowska, N 1912

Tabata, T 1216

Tabernero, J 1607

Tabori, U 1129

Tada, H 852

Tafas, T 789

Tagg, SLC 1842

Taillade, L 1380

Tait, D 695

Tajiri, T 350

Takada, K 1816

Takano, S 305

Takemasa, I 1307

Takeno, A 1307

Takeshima, N 1216

Takiguchi, S 1307

Takizawa, K 1216

Talieri, M 2094

Tallini, G 83

Talvinen, K 335

Tamaya, T 1557

Tan, HK 357

Tanaka, N 1468

Tanaka, YO 1034

Tang, Z 911

Tannapfel, A 78

Tapio, S 1089

Tariverdian, M 1867

Tarrier, N 1794

Tassi, RA 768

Tatarov, O 1769

Taubert, H 1083

Taylor, MB 591

Taylor, NJ 321

Taylor, P 442

Teixeira, AS 978

Tekle, C 750

Tellez, C 1265

Temam, S 357

ten Hoor, KA 341

Teng, L 491

Tepper, J 2001

Terracciano, L 1712

Terry, SYA 670

Testa, E 1402

Thadhani, R 1161

Thall, PF 1426

Thatcher, N 442

Theodore, C 1959

Thomas, H 1726

Thomas, MB 722

Thommesen, L 1330

Thompson, AM 1763

Thompson-Fawcett, M 966

Thomsen, HF 1522

Thorne, N 663

Thureau, S 1586

Thurston, G 1204

Tighiouart, M 1684

Tijssen, M 1802

Tillier, CN 259

Timms, JF 679

Timotheadou, E 1775

Tirelli, U 1364

Tobelem, G 1555

Todo, S 1891

Toida, M 647

Tokino, T 384

Tölle, A 939

Toloczko-Grabarek, A 974

Tomlinson, IPM 1726, 2088

Tong, W-G 1064

Tong, X-J 1656

Tonini, G 716, 1402

Tootell, S 569

Torri, V 51

Torrisi, R 1564

Tortora, G 473

Toschi, L 83

Toubanakis, C 72

Tougeron, D 1586

Tovey, S 1769

Toyota, M 384

Tozer, G 321

Treeck, O 1246

Trepo, C 143

Tretli, S 1165

Trichopoulos, D 191, 1544

Trichopoulou, A 191

Trinh, BX 1735

Tristram, A 1929

Troisi, R 1161

Trypaki, M 923

Tschense, A 1946

Tsipouras, P 789

Tsuboi, M 852

Tsuda, H 1651

Tsuji, I 176, 1179, 1502

Tsuji, T 408

Tsukui, T 350

Tsuruo, T 283

Tudur Smith, C 6

Tuikkala, J 335

Tulman, A 371

Turner, AJ 1114

Turner, G 1383

Turney, BW 789

Tworoger, SS 1916

Tyasmorowati, D 214

Tyndel, S 1007

Udelnow, A 1083

Ueda, M 655

Ulbrich, K 900

Umekita, Y 408

Uno, K 1034

Usmani, BA 1114

Valik, D 1103

Valle, JW 577

van Breda, E 30

van Dam, PA 1735

van de Velde, CJH 1802

van den Akker, BEWM 214

Van den Bosch, SM 1735

van den Eertwegh, AJM 259

Van den Eynden, GG 1735

Van der Auwera, I 1735

van der Graaf, WTA 1600

van der Hel, O 1912

Van der Straaten, T 275, 1316

van der Veldt, AAM 259

van der Zee, AGJ 341

van Engeland, M 727, 1735

van Grieken, NCT 1802

van Helvoort, A 2029

Van Laere, SJ 1735

Van Marck, EA 1735

van Norren, K 2029

Van Orden, KL 100

van Rij, A 966

van Wouwe, M 259

Vanky, E 201

Varella-Garcia, M 83

Vasconcelos, GM 1668

Vassileva, V 2037

Vatten, LJ 201

Veal, GJ 894

Veerakumarasivam, A 663

Venkatraman, G 949

Vergini, V 239

Verhagen, CAH 1408

Vermeulen, PB 1735

Versmold, B 974

Vet, JNI 214

Viale, G 1564

Vichodez, G 371

Vickaryous, N 1726

Vilar, E 1607

Vilholm, OJ 604

Villanueva, A 1718

Vincent, T 219

Vincenzi, B 716, 1402

Violet, JA 632

Viret, F 1395

Vitvitski, L 143

Voelker, B 2065

Vogt, U 774

Voigt, J-J 1959

von Knebel Doeberitz, M 1867

von Mehren, M 1415

Von Pawel, J 2006

Vorsa, N 1823

Vos, P 2029

Voutsina, A 923

Vreugdenhil, G 30

Waddell, T 577

Wager, M 1153

Wagner, T 371

Wagner, W 622

Wakeford, R 1194

Wakelam, MJO 556

Walch, AK 1089

Waldmann, J 1900

Walker, M 711, 1572

Wallard, MJ 663

Waller, M 1991

Walsh, L 1946

Wands, JR 143

Wang, A 774

Wang, B 68

Wang, BS 811

Wang, R 196, 1511

Wang, X 789

Wang, Y 911, 1656

Wang, YZ 464

Wang, Z 1695

Wangsa, D 1121

Ward, TH 841

Wardley, AM 597

Warner, B 72

Warner, E 371

Wassermann, K 939

Watanabe, H 350

Watanabe, M 1462

Watson, E 1007

Waugh, DJJ 2054

Weaver, MA 23

Webb, E 2088

Webster, L 989

Wehrum, D 1484

Wei, W 1136

Weinstock, RM 545

Weitzel, J 371

Wellsted, D 321

Wen, S 1426

Wentzensen, N 1867

Wenzel, S 930

West, R 1786

Westwood, FR 1256

Wheeler, TE 1330

Whelan, J 1967

Whynes, DK 1991

Whyteside, AR 1114

Wiedenmann, B 110

Wiemels, JL 1668

Wight, E 428

Wilding, JL 839

Williams, MV 695

Wilson, BC 2037

Wilson, C 704, 1013, 2054

Wilson, CL 383

Wilson, G 577

Wilson, MS 591

Wilson, RH 2054

Wilson, S 556

Wirtz, RM 1775

Wisman, GBA 341

Witham, G 591

Witney-Smith, C 72

Witton, CJ 1296

Wölber, L 1269

Wolfert, RL 1103

Wolff, RA 722

Wolin, KY 995

Wolter, P 448

Wong, AST 283

Wong, CKC 283

Wong, K-K 245

Wong, KY 283

Wong, V 1129

Wong, YC 1832

Wong, YP 2083

Worthington, J 2054

Wotherspoon, A 868

Wouters, BG 727, 1884

Wrba, F 151

Wu, AH 196

Wu, C 911

Wu, Y 1572

Würl, P 1083

Wyatt, JC 415

Xie, D 1656

Xu, Z 1684

Xynopoulos, D 2094

Yagüe, E 387

Yamamoto, N 1757

Yamamoto, Y 655

Yamaoka, H 1891

Yamasaki, M 1307

Yamashita, T 647

Yan, H 1179

Yang, C-P 23

Yannoukakos, D 974

Yao, J 1695

Yap, WN 1832

Yap, YL 1832

Yasmeen, A 404

Yasui, H 1442

Yataghene, Y 1395

Ychou, M 1395

Ye, W 527

Yeap, BY 245

Yeasmin, S 2020

Yeh, C-H 23

Yeoh, K 1678

Yeung, BHY 283

Yi, SY 167

Yilmaz, M 858

Yokomizo, T 1064

Yoon, H-S 966

Yoshida, S 1232

Yoshidome, H 305

Yoshikawa, H 1034

Yoshikawa, R 1196

Yoshinaka, H 408

Yoshitomi, H 305

Yu, H 1357, 1695

Yu, J 2083

Yu, MC 196, 1511

Yung, A 781

Zabinski, RF 100

Zanetti-Dällenbach, R 428

Zang, DY 584

Zanier, L 239

Zappa, M 239, 423, 1756

Zarcone, M 423, 1756

Zaridze, D 1912

Zatloukal, K 151

Zatovicova, M 1348

Zeisberg, E 1375

Zetterberg, A 513

Zhang, H 536, 1684

Zhang, X 1684

Zhao, L 1695

Zhao, X 245

Zheng, T 1934

Zheng, Y 1103

Zhou, G 513

Zimmermann, F 1020

Zlobec, I 1712

Zografos, G 1775

Zorzi, M 423, 1756

Zucali, PA 51

Zwickl, H 151

